# Laboratory predictors of sickle cell disease severity: a cross-sectional study

**DOI:** 10.25122/jml-2023-0397

**Published:** 2024-01

**Authors:** Mortadah Alsalman

**Affiliations:** 1 Department of Medicine, College of Medicine, King Faisal University, Alahsa, Saudi Arabia

**Keywords:** sickle cell disease, thrombocytosis, leukocytosis

## Abstract

Sickle cell disease (SCD) is the most common monogenic disorder, although the diversity and heterogenicity of clinical presentations render estimations of disease severity unpredictable. This cross-sectional study aimed to determine if laboratory markers could serve as indicators of SCD severity. We enrolled 90 adult patients with SCD with a mean age of 32.33 ± 11.84 years from the eastern province of Saudi Arabia, where SCD is more common than in other regions. Our study revealed a positive significant association between the number of hospitalizations and emergency visits with white blood cells (WBC) (R = 0.241, R = 0.207), respectively. Similarly, positive significant associations were found between the number of hospitalizations and emergency visits with platelets (R = 0.393, R = 0.276), respectively. Conversely, negative significant relationships were found between the number of hospitalizations and emergency visits (ER) with hemoglobin (Hb) F (R = -0.268, R = -0.263), respectively. Additionally, significant negative relationships were found between Hb F (R = -0.223) and the frequency of ICU admission. Only the number of hospitalizations and emergency visits annually were significantly predicted with *P* values of 0.021 and 0.038, respectively. Moreover, an increase in WBC was found to significantly increase the chance of undergoing splenectomy by 23.02%. SCD is a multisystemic disease with heterogeneous clinical presentations and disease severity. Inflammatory markers are valuable tools for better risk stratification and could be translated into developing new therapeutic strategies and modifying the treatment paradigm.

## INTRODUCTION

Sickle cell disease (SCD) is an autosomal recessive disorder caused by the inheritance of abnormal beta-globin alleles carrying the sickle mutation on the beta-globin (HBB) gene. It is the most common monogenetic disease, with millions affected worldwide [1,2]. In the United States, around two million individuals are carriers, with approximately 72,000 experiencing this condition. According to the Saudi Premarital Screening Program, sickle cell gene prevalence is estimated to be 4.2% for sickle cell trait and 0.26% for SCD in the adult population. The Eastern province has the highest prevalence rates, approximately 17% for sickle cell trait and 1.2% for SCD [3,4].

Pain is the hallmark of SCD that increases throughout life, leading to hospitalization and poor quality of life. Nevertheless, SCD is a multisystem complex disorder that is also characterized by chronic hemolytic anemia, progressive organ failure, and increased risk of mortality [5,6]. In the 1970s, the median survival rate for patients with sickle cell disease was around 20 years. However, with the introduction of measures such as antibiotic prophylaxis to combat encapsulated organisms, vaccinations, and the use of hydroxyurea, there has been a significant improvement in survival rates for SCD patients [6,7]. However, survival has not changed over the last 25 years, and multiple studies indicate that certain groups of patients are at high risk of early mortality compared to others. For instance, adults with a tricuspid regurgitant velocity (TRV) of at least 2.5 m/s, obstructive pulmonary disease, and chronic kidney disease die early [8,9]. The diversity and heterogenicity of SCD clinical presentations render estimations of disease severity unpredictable. Additionally, there is limited data addressing predictors of SCD severity [7,10]. Therefore, this study aimed to investigate whether laboratory indices could be utilized as predictors of SCD severity.

## MATERIAL AND METHODS

This study was conducted in August 2023, in the eastern region of Saudi Arabia, where sickle cell disease is more widespread in comparison to other areas. Participants were adult Saudi patients with SCD, aged 18 to 65, selected through a systematic non-randomized sampling method. The researchers provided the participants with a comprehensive explanation of the study objectives, the data collection process, the significance of conducting this research, the right to participate, and the confidentiality and anonymity of the data.

Each participant was interviewed by a trained interviewer to complete a survey. The survey consisted of demographic and clinical information, including comorbidities, frequency of hospitalizations and emergency visits, blood transfusion, previous intensive care unit (ICU) admission, history of stroke, and surgical and medication history.

Furthermore, additional clinical and laboratory data, including complete blood count, hemoglobin electrophoresis, lactate dehydrogenase (LDH), and kidney function tests, were obtained via the electronic database of the hospital.

Descriptive statistics were computed and reported as frequencies and proportions (%) for categorical variables, while mean and standard deviation were used to describe continuous variables. The severity indicators and predictors were evaluated using a Pearson correlation. The strength of the association was assessed using Cohen's standard, where coefficients between 0.10 and 0.29 indicated a small effect size, coefficients between 0.30 and 0.49 indicated a moderate effect size and coefficients above 0.50 indicated a large effect size. The results were statistically significant at *P* <0.05. The data was analyzed using the Statistical Package for Social Sciences (SPSS) Version 26, developed by IBM Corporation in Armonk, New York.

## RESULTS

This study interviewed ninety adult patients with sickle cell disease with a mean age of 32.33 ± 11.84 years. The mean number of ICU admissions was 2.75 ± 3.01, and the mean number of emergency visits per year was 8.43 ± 5.61. Patient demographics and characteristics are shown in Table 1.

**Table 1:** Clinical data of participants (*n* = 90)

Characteristics	No.	%
**Age (Mean ± SD)**	**32.33 ± 11.84**	
**Gender**		
Male	27	30
Female	63	70
**Educational level**		
No formal education	5	5.5
Elementary school	11	12.2
Intermediate school	16	17.8
Secondary higher educations	38	42.2
Graduation	17	18.9
Master	3	3.3
**Previous ICU admissions**		
Yes	67	74.4
No	23	25.6
**History of stroke**		
Yes	1	1.1
No	89	89.9
**Splenectomy**		
Yes	13	14.4
No	77	58.6
**Cholecystectomy**		
Yes	33	36.7
No	57	63.3
**Joint replacement**		
Yes	5	5.6
No	85	94.4
**Have you been started on hydroxyurea?**		
Yes	42	46.7
No	48	53.3
**Number of blood transfusion throughout life (Mean ± SD)**	6.29 ± 4.3	
**Frequency of ICU admission (Mean ± SD)**	2.75 ± 3.01	
**Number of hospitalizations per year (Mean ± SD)**	4.7 ± 4.57	
**Number of emergency visits per year (Mean ± SD)**	8.43 ± 5.61	

Our study revealed a positive significant relationship between the number of hospitalizations and emergency visits with the white blood cell (WBC) (R = 0.241, R = 0.207), respectively. Similarly, positive significant associations were found between the number of hospitalizations and emergency visits with platelets (R = 0.393, R = 0.276), respectively. Conversely, negative significant relationships were found between the number of hospitalizations and emergency visits (ER) with hemoglobin (Hb) F (R = -0.268, R = -0.263), respectively. Additionally, significant negative relationships were found between Hb F (R = -0.223) and the intensive care unit admission frequency.

Multiple linear regression analyses were conducted to predict the frequency of hospitalization, ER visits, blood transfusions, and ICU admission. Only the number of hospitalizations and emergency visits annually were significantly predicted with *P* values of 0.021 and 0.038, respectively (Tables 2 and 3). On the other hand, the models intended to predict the number of blood transfusions and frequency of ICU admission were not significant (*P* values of 0.055 and 0.075), respectively. Furthermore, binary logistic regression analyses were conducted to predict joint replacement and splenectomy. The prediction of joint replacement was not significant (*P* = 0.608), while the model for splenectomy prediction was significant (*P* <0.001). An increase in WBC was significantly associated with an increased likelihood of splenectomy, where a one-unit increase in WBC raised the chances of undergoing a splenectomy by 23.02% (Table 4).

**Table 2:** Linear regression predicting the number of hospitalizations per year

Variable	B	SE	95.00% CI	β	t	*P*
**(Intercept)**	-7.57	18.04	[-43.47, 28.34]	0.00	-0.42	.676
**WBC**	0.06	0.11	[-0.15, 0.27]	0.07	0.56	.576
**HbA**	0.09	0.18	[-0.27, 0.46]	0.34	0.51	.614
**Platelets**	0.007	0.003	[0.001, 0.01]	0.29	2.46	.016
**LDH**	0.001	0.003	[-0.006, 0.008]	0.04	0.36	.720
**Gender**	-0.83	1.06	[-2.93, 1.27]	-0.08	-0.79	.435
**HbF**	-0.04	0.17	[-0.37, 0.29]	-0.06	-0.26	.798
**HbS**	0.10	0.19	[-0.28, 0.48]	0.32	0.53	.599
**MCV**	0.03	0.04	[-0.06, 0.11]	0.07	0.62	.534

B, Unstandardized Beta; SE, Standard Error; CI, Confidence Interval; β, Standardized Beta; t, t-Statistic; WBC, White Blood Cell count; HbA, Hemoglobin A; HbF, Hemoglobin F; HbS, Hemoglobin S; MCV, Mean Corpuscular Volume; LDH, Lactate Dehydrogenase.

**Table 3:** Linear regression predicting the number of emergency visits per year

Variable	B	SE	95.00% CI	β	t	*P*
**(Intercept)**	-2.60	22.36	[-47.09, 41.88]	0.00	-0.12	.908
**WBC**	0.08	0.13	[-0.19, 0.34]	0.07	0.57	.572
**HbA**	0.07	0.23	[-0.39, 0.52]	0.19	0.29	.775
**Platelets**	0.004	0.004	[-0.004, 0.01]	0.12	1.00	.322
**MCV**	0.05	0.05	[-0.06, 0.15]	0.10	0.90	.369
**HbF**	-0.11	0.20	[-0.51, 0.30]	-0.13	-0.51	.609
**HbS**	0.03	0.24	[-0.44, 0.50]	0.08	0.13	.894
**LDH**	0.004	0.004	[-0.004, 0.01]	0.12	1.05	.298
**Gender**	1.58	1.31	[-1.02, 4.18]	0.13	1.21	.231

B, Unstandardized Beta; SE, Standard Error; CI, Confidence Interval; β, Standardized Beta; t, t-Statistic; WBC, White Blood Cell count; HbA, Hemoglobin A; MCV, Mean Corpuscular Volume; HbF, Hemoglobin F; HbS, Hemoglobin S; LDH, Lactate Dehydrogenase.

**Table 4:** Binary logistic regression predicting splenectomy

Variable	B	SE	χ^2^	*P*	OR	95.00% CI
**(Intercept)**	-5.01	48.42	0.01	.918	-	-
**WBC**	0.21	0.10	4.47	.034	1.23	[1.02, 1.49]
**HbA**	-0.004	0.52	0.00	.994	1.00	[0.36, 2.74]
**platelets**	0.005	0.002	3.31	.069	1.00	[1.00, 1.01]
**MCV**	0.02	0.05	0.19	.661	1.02	[0.93, 1.12]
**HbF**	-0.17	0.48	0.12	.732	0.85	[0.33, 2.19]
**HbS**	0.04	0.52	0.01	.940	1.04	[0.37, 2.89]
**LDH**	-0.006	0.004	2.31	.128	0.99	[0.99, 1.00]
**Gender**	-1.15	1.08	1.13	.287	0.32	[0.04, 2.62]

B, Unstandardized Beta; SE, Standard Error; CI, Confidence Interval; β, Standardized Beta; t, t-Statistic; WBC, White Blood Cell count; HbA, Hemoglobin A; MCV, Mean Corpuscular Volume; HbF, Hemoglobin F; HbS, Hemoglobin S; LDH, Lactate Dehydrogenase.

## DISCUSSION

Sickle cell disease is a complex disorder that reflects underlying complex pathophysiology. Despite the existence of several disease-modifying agents, there are still unmet needs. Stem cell transplantation and gene therapy are attractive potential curative treatment options for SCD but are faced with some limitations and potential complications. These include lack of matched siblings, graft rejections, and possible risk of secondary malignancies [11,12]. Therefore, risk stratifications for patients with SCD might help identify individuals who could benefit from these treatments or interventions at earlier stages in their lives. Several models for classification systems in clinical settings, such as the New York Heart Association (NYHA) classification, Confusion, Urea, Respiratory rate, Blood pressure, 65 years of age and older (CURB-65) for pneumonia, or the Acute Physiology and Chronic Health Evaluation II (APACHE II) for sepsis, have been developed and widely used due to their validity, reliability, and clinical value [13]. Although there is no such classification system for SCD, there have been previous great efforts to establish severity indexes for patients with SCD. For instance, Shah and colleagues, in their recent study, devised a system that categorizes patients into homogeneous groups based on disease state. This system incorporates various factors, including organ damage, hemoglobin genotype, age, presence of chronic pain, and the frequency of unscheduled acute care visits per year. The purpose of this system is to aid clinical decision-making by providing a more comprehensive understanding of patient characteristics [13]. However, further studies are still needed to validate this system.

This study was designed to investigate laboratory markers that could predict SCD severity. It showed that patients with leukocytosis tend to have frequent hospitalization as well as increased emergency visits, irrespective of the severity of the crisis. Interestingly, leukocytosis was more prevalent among those who underwent splenectomy. However, our study is retrospective, and we cannot assess whether participants had leukocytosis before splenectomy or as a consequence. These findings add to previous reporting that leukocytosis among patients with SCD is not restricted to poor outcomes but also to frequency of visits, which translates into poor quality of life and increased economic burden [14,15]. Similarly, thrombocytosis was significantly correlated with annual hospitalization and frequent emergency visits but poorly correlated with intensive care unit admissions. These are consistent with reports indicating thrombocytopenia is more common in individuals with multiorgan failure and non-survivors [16,17]. On the other hand, the level of hemoglobin F was inversely proportional to the frequency of hospitalization and ICU admission. In other words, the higher the hemoglobin F, the lower the severity of the disease, which is consistent with the fact that hemoglobin F is known to ameliorate the clinical complications of SCD [18].

This study emphasizes that the inflammatory process of SCD represented by leukocytosis and thrombocytosis plays a vital role in determining disease severity but does not necessarily predict poor outcomes and mortality. Additionally, persistent steady-state leukocytosis and thrombocytosis highlight that inflammation is an ongoing process with inflammation resolution failure [19]. Contrarily, a high hemoglobin F level is a helpful predictor and modifier of SCD severity, though none of these markers could predict individuals with increased demand for frequent blood transfusion. However, these factors should be incorporated into a risk stratification system to recognize individuals with severe disease and offer them curative treatment or use a multimodal approach at the early stage of the disease. Furthermore, additional studies are needed to identify more predictors of morbidity and whether these markers could be used to predict mortality.

## CONCLUSION

SCD is a multisystemic disease with heterogeneous clinical presentations and disease severity. Inflammatory markers are valuable tools for better risk stratification and could be translated into developing new therapeutic strategies and modifying the treatment paradigm.
